# Epigenetic silencing of TET2 and TET3 induces an EMT-like process in melanoma

**DOI:** 10.18632/oncotarget.13324

**Published:** 2016-11-12

**Authors:** Fuxing Gong, Yu Guo, Yiqian Niu, Jiawei Jin, Xiaojuan Zhang, Xiaoqian Shi, Limeng Zhang, Runting Li, Longxin Chen, Runlin Z. Ma

**Affiliations:** ^1^ State Key Laboratory for Molecular Developmental Biology, Institute of Genetics and Developmental Biology, Chinese Academy of Sciences, Beijing 100101, China; ^2^ Zhengzhou City Key Laboratory of Molecular Biology, Zhengzhou Normal University, Zhengzhou 450044, China; ^3^ University of the Chinese Academy of Sciences, Beijing 100101, China

**Keywords:** TGF-β, TET, DNMT3A, EMT, Melanoma

## Abstract

Epithelial-Mesenchymal Transition (EMT) is a critical step in the progression of cancer. Malignant melanoma, a cancer developed from pigmented melanocytes, metastasizes through an EMT-like process. Ten-eleven translocation (TET) enzymes, catalyzing the conversion of 5-methylcytosine (5mC) to 5-hydroxylmethylcytosine (5-hmC), are down regulated in melanoma. However, their roles in the progression and the EMT-like process of melanoma are not fully understood. Here we report that DNA methylation induced silencing of *TET2* and *TET3* are responsible for the EMT-like process and the metastasis of melanoma. *TET2* and *TET3* are down regulated in the TGF-β1-induced EMT-like process, and the knocking down of *TET2* or *TET3* induced this EMT-like process. A DNA demethylating agent antagonized the TGF-β-induced suppression of TET2 and TET3. Furthermore, a ChIP analysis indicated that enhanced recruitment of DNMT3A (DNA Methyltransferase 3A) is the mechanism by which TGF-β induces the silencing of *TET2* and *TET3*. Finally, the overexpression of the TET2 C-terminal sequence partially rescues the TGF-β1-induced EMT-like process *in vitro* and inhibits tumor growth and metastasis *in vivo*. Hence, our data suggest an epigenetic circuitry that mediates the EMT activated by TGF-β. As an effector, DNMT3A senses the TGF-β signal and silences TET2 and TET3 promoters to induce the EMT-like process and metastasis in melanoma.

## INTRODUCTION

Malignant melanoma is a cancer that develops from pigmented melanocytes. Although it does not originate from epithelial cells, the hallmarks of Epithelial-Mesenchymal Transition (EMT) play critical roles in its progression [1]. Two transcriptional signatures of the melanoma cell lines were identified as respectively proliferative and invasive cellular phenotypes [2]. Following the primary lesion formation by proliferative cells, alternations of the micro-environmental conditions give rise to cells with the invasive signature through an EMT-like process. When these invasive cells reach suitable distal sites, they can return to the proliferative state. Through such a proliferative-invasive-proliferative cycle, melanoma cells can metastasize repeatedly [1].

EMT is a developmental process that enables epithelium originated cells to gain mesenchymal-like properties, including loss of apical-basal polarity and cell-cell adhesion, switch of cell surface markers and enhanced migratory and invasive capacities [3]. The reprogramming of gene expression during EMT is driven by ZEB (zinc finger E-box binding), Snail and bHLH (basic helix-loop-helix) family of transcription factors, which all inhibit E-cadherin transcription [4].

Epigenetic regulators have been suggested to be involved in the regulation of the EMT. For example, the miR-200 family members, suppressors of EMT, are silenced through DNA methylation [5]. AID (Activation-induced cytidine deaminase), which is involved in DNA demethylation, has been reported to be critical for the EMT induced by inflammatory signals in mammary epithelial cells [6]. Additionally, the epigenetic silencing of SOX9 (SRY-Box 9) is responsible for the invasiveness of melanoma [7].

Transforming growth factor-β (TGF-β), an effective inducer of EMT, is frequently used for the activation of EMT in a variety of epithelial cells [8]. It binds to the types I and II receptor serine/threonine kinases and activates the Smad complexes to regulate gene transcription [9]. The disruption of Smad signaling in basal-like breast cancer cells impairs the DNA methylation profile and activates the expression of many silenced genes which accompanied an MET (Mesenchymal-Epithelial Transition) property [10, 11]. This suggests that through its involvement in TGF-β signaling, the DNA methylation status plays a critical role in the maintenance of the invasiveness of tumors.

The DNA methylation profile of a cell is maintained by both the DNA methylation and demethylation pathways. DNA methylation occurs as the transfer of a methyl group onto the cytosine of the CpG dinucleotides by DNA methyltransferases [12]. DNA demethylation occurs either passively or actively. Active DNA demethylation refers to the process by which enzymes catalyze the removal of a methyl group from 5-methylcytosine (5mC). One pathway is the oxidation process in which TET (Ten-eleven translocation) enzymes catalyze the conversion of 5mC to 5-hydroxylmethylcytosine (5-hmC) [13]. The second pathway involves the deamination pathway catalyzed by the AID/APO (Apo-lipoprotein B) complex [14, 15]. Passive DNA demethylation occurs when DNA methyltransferases are inhibited [13]. Because aberrant DNA methylation is a prominent feature of cancer cells [16], DNA methylation or demethylation pathways may contribute to cancer progression. The progressive loss of 5-hmC and the down regulation of TET proteins are reported to be associated with the progression from benign “nevus” to aggressive melanomas [17, 18]. Loss of 5-hmC is therefore suggested as a marker to distinguish between benign cells and malignant melanomas [19].

Given the increasing evidence that shows the importance of the regulation of DNA methylation in cancer progression, we hypothesized that TET proteins play critical roles in the EMT-like process of melanoma. We found decreased expression of TET2 and TET3 in the TGF-β1-induced EMT-like process, and shRNA-mediated knockdown of *TET2* or *TET3* induced the EMT-like process and up regulated the expression of several critical EMT transcriptional regulators. Mechanistically, TGF-β1 stimulated endogenous DNMT3A/3B expression, while depletion of DNMT3A activated *TET2* and *TET3* expression. ChIP-PCR experiment further showed that the recruitment of DNMT3A to *TET2* and *TET3* promoters was enhanced by TGF-β treatment. Moreover, the overexpression of the TET2 C-terminal domain partially suppressed the TGF-β1-induced EMT-like process *in vitro* and inhibited tumor growth and metastasis *in vivo*. Taken together, our results indicate that TET2 and TET3 are suppressors of the EMT-like process in melanoma and that the silencing of these proteins by DNA methylation is one mechanism by which TGF-β induces an EMT-like process.

## RESULTS

### TET2 and TET3 are down regulated in the EMT-like process of melanoma cells

**Figure 1:**
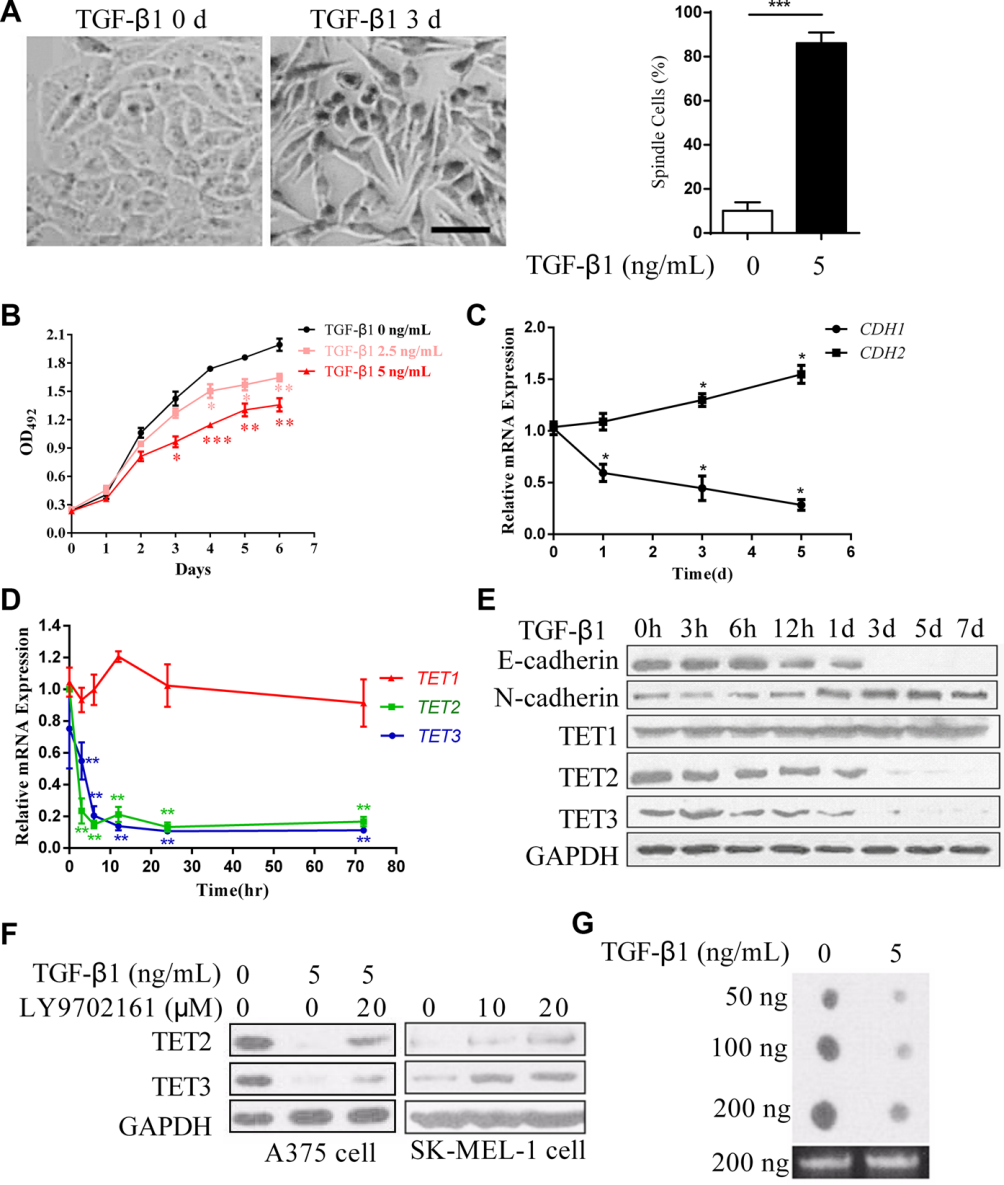
Down regulation of TET2 and TET3 in the TGF-β1-induced EMT-like process in A375 cells (**A**) Representative morphologic changes of A375 cells cultured with or without TGF-β1 (5 ng/ml) for 3 days were shown on the left, Scale bar = 50 μm, the percentage of spindle cells were shown on the right; (**B**) The effect of TGF-β1 on cell growth was evaluated by measuring the absorbance of OD_492_ after staining with MTT; (**C**) and (**D**) Real-time RT-PCR analysis of the relative expression levels of *CDH1*, *CDH2* and *TET1/2/3* mRNAs in A375 cells stimulated with 5 ng/ml TGF-β1 for the indicated times. The data are displayed as the fold change in the expression levels in untreated cells; the levels were normalized to those of *GAPDH*, and the error bars represent the mean ± SD of triplicate experiments (Student's *t-test*, *p < 0.05, **p < 0.01); (**E**) Expression of E-cadherin, N-cadherin and TET1/2/3 in A375 cells cultured with 5 ng/ml TGF-β1 for the indicated times was examined by immunoblotting. GAPDH was used to show that equal amounts of proteins were loaded on the gel; (**F**) Immunoblotting analysis of endogenous TET2 and TET3 expression levels in A375 cells and SK-MEL-1 cells that were treated with vehicle (0), the TGF-β type I receptor inhibitor LY9702161 or that were stimulated with TGF-β1 for 3 days, GAPDH was used to show that equal amounts of proteins were loaded on the gel; (**G**) The 5hmC levels of the genome of the cells treated with or without TGF-β were detected by dot-blotting.

TGF-β is an effective inducer of EMT that facilitates tumor metastasis [20]. A375 cells, derived from human melanoma, typically exhibit a cobblestone-like morphology with tight cell adhesions. The 3 days' treatment of TGF-β1 endowed the cells with an elongated fibroblast-like morphology and a scattered distribution (Figure 1A). And the proliferation of the cells was significantly inhibited (Figure 1B). A switch from E-cadherin to N-cadherin is frequently used as a marker of EMT [4], and, as expected, the down regulation of E-cadherin (*CDH1*) and the up regulation of N-cadherin (*CDH2*) occurred at both mRNA and protein levels (Figure 1C and 1E). These data indicate that TGF-β1 induces an EMT-like process. Intriguingly, both TET2 and TET3, but not TET1, were significantly decreased, as shown by quantitative real-time RT-PCR and immunoblotting, respectively (Figure 1D and 1E). Similar results were observed in M619 cells and SK-MEL-28 cells (Supplementary Figure S1). In another experiment, the down regulation of TET2 and TET3, induced by TGF-β1 was rescued by LY9702161, a TGF-β type I/II receptor small molecule inhibitor (Figure 1F). Treatment of the metastatic melanoma cell line SK-MEL-1, which expressed very low levels of TET2 and TET3 (Supplementary Figure S2), with LY9702161, elevated their expression levels (Figure 1F). TET proteins are responsible for the generation of 5hmC of the genome, we assessed the 5hmC levels of the cells, and found that the treatment of TGF-β1 decreased the 5hmC levels of the cells (Figure 1G). These data indicate that TET2 and TET3 are potential suppressors of the EMT-like process.

### Knockdown of TET2 or TET3 induces an EMT-like process

**Figure 2:**
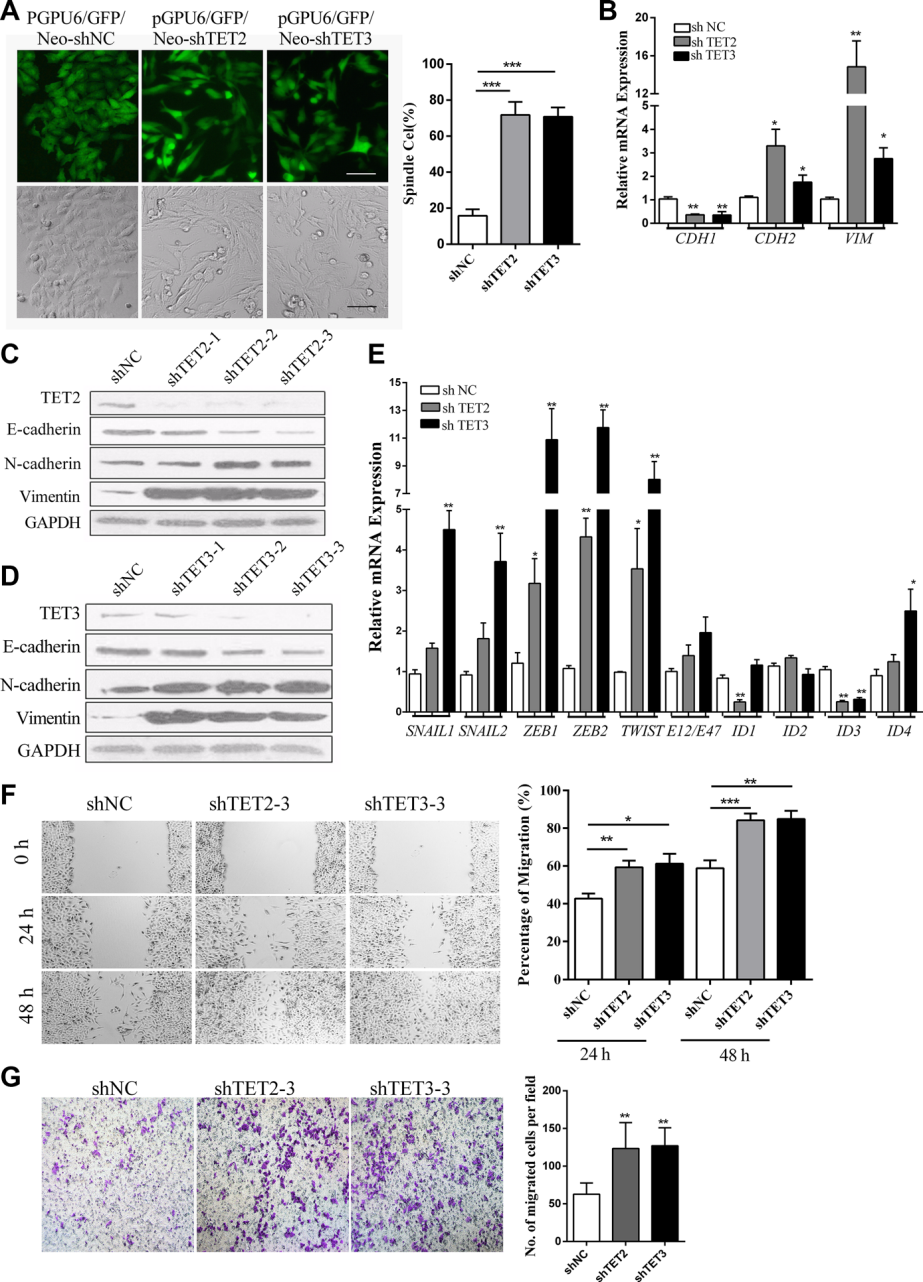
Knock down of TET2 or TET3 induces an EMT-like Process (**A**) Morphological changes in A375 cells induced by the shRNA-mediated knock down of TET2 or TET3 were observed by optical microscopy and fluorescence microscopy, the cells that were transfected with shRNAs carrying GFP-coding genes were selected by G418 for 2 weeks, and single clones were photographed and shown on the left, Scale bar = 50 μm, the percentage of spindle cells were shown on the right; (**B**), (**C**) and (**D**) E-cadherin (*CDH1*), N-cadherin (*CDH2*) and Vimentin (*VIM*) mRNA and protein expression levels in A375 cells transfected with shRNAs against TET2 or TET3 were assessed by real-time RT-PCR (B) and immunoblotting (C)(D). For RT-qPCR, the relative expression levels of all genes were normalized to *GAPDH* (Student's *t-test*, *p < 0.05, **p < 0.01), and for immunoblotting, GAPDH was used to show that equal amounts of proteins were loaded on the gel; (**E**) The relative mRNA expression levels of EMT master transcription factors were detected by real-time RT-PCR and were normalized to *GAPDH* (Student's *t-test*, *p < 0.05, **p < 0.01); (**F**) For the wound healing assay, the cells were grown to confluence in complete cell culture medium. At time 0, a 3-mm scrape wound was created across the diameter with a pipette tip followed by extensive washes with medium to remove dead and floating cells. Cell migration was determined by measuring the distance between the cells on either side of the scratch over 24 hrs and 48 hrs, which is shown on the right (**p < 0.01, compared with the control). Representative wound closure was monitored by microscopy at × 100 magnification and is shown on the left; (**G**) For the Boyden Chamber Transwell cell migration assay, 5 × 10^6^ cells were seeded on top of the Boyden chambers. After 24 hrs, the cells on the bottom were stained with 1% crystal violet and were observed by optical microscopy. The migrated cells were counted and representative images of the migrated cells are shown on the right; the Y-axis represents the stained cell counts per field. The data represent three independent experiments. (Student's *t-test*, *p < 0.05, **p< 0.01).

The down regulation of TET2 and TET3 induced by TGF-β1 suggested their possible roles in the induction of the EMT-like process. We proceed to establish stable A375 cell clones in which TET2 or TET3 was knocked down by shRNAs. Morphologically, the cells that were transfected with TET2 or TET3-targeted shRNAs exhibited prominent mesenchymal-like phenotypes, including gain of a fibroblast-like morphology and loss of the majority of cell-cell contacts (Figure 2A). The knock down of TET2 or TET3 induced a switch from E-cadherin to N-cadherin of the cell surface proteins and activated Vimentin expression (Figure 2B, 2C and 2D). EMT master transcription factors, including *SNAIL1*, *SNAIL2*, *ZEB1*, *ZEB2*, *TWIST*, and *ID4* were found to be transcriptionally up regulated (Figure 2E). Moreover, the cells displayed higher motility in a wound healing assay (Figure 2F) and faster migration in a Boyden Chamber assay (Figure 2G). These results suggest that the knockdown of TET2 or TET3 induces an EMT-like process, which might promote the motility of melanoma cells through the regulation of their intrinsic metastatic capability.

### Methylation of CpG islands is involved in TGF-β-induced down regulation of TET2 and TET3

**Figure 3:**
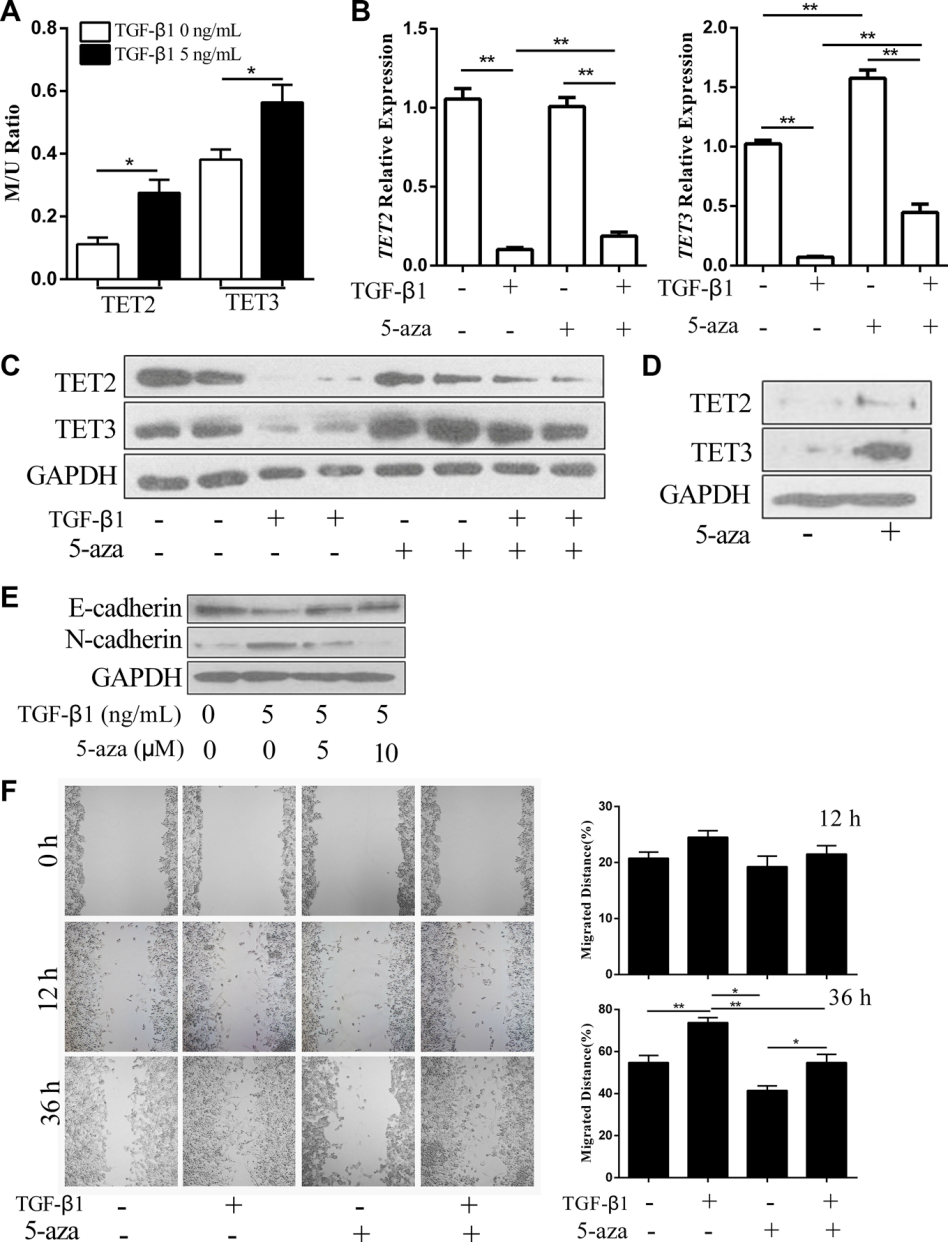
5-aza antagonizes the TGF-β1-induced suppression of TET2 and TET3 and the EMT-like process (**A**) The relative methylation levels of the TET2 and TET3 promoters after treatment with or without TGF-β1 were analyzed by methylation- and non-methylation-specific qPCR. The Y-axis represents the relative methylation levels normalized by the non-methylation levels (Student's *t* test, *p < 0.05); (**B**) (**C**) RT-qPCR (B) and immunoblotting (C) analysis of TET2 and TET3 mRNA and protein expression levels in A375 cells treated with or without TGF-β1 and 5-aza; for RT-qPCR, the relative expression levels of all genes were normalized to the *GAPDH* level (Student's *t* test, *p < 0.05, **p < 0.01), while for immunoblotting, GAPDH was used to show that equal amounts of proteins were loaded on the gel; (**D**) The expression levels of TET2 and TET3 in SK-MEL-1 cells treated with or without 5-aza were analyzed by immunoblotting, and GAPDH was used to show that equal amounts of proteins were loaded on the gel; (**E**) The expression of E-cadherin and N-cadherin in A375 cells treated with or without TGF-β1 or 5-aza were analyzed by immunoblotting, and GAPDH was used to show that equal amounts of proteins were loaded on the gel; (**F**) Wound healing assay using A375 cells treated with or without TGF-β1 and 5-aza. Representative images of migrated cells are shown on the left. The mean was derived from cell counts of 4 fields, and each experiment was repeated 3 times (Student's *t* test, *p < 0.05, **p < 0.01, compared with the control).

The methylation on gene promoters usually leads to gene silencing [13]. A screen of *TET2* and *TET3* gene sequences led to the identification of CpG islands near their transcription start sites. Therefore, we hypothesized that TGF-β1 may suppress TET2 and TET3 expression through the induction of DNA methylation of their promoters. Methylation-specific qPCR analysis indicated that TGF-β1 treatment elevated the methylation level of their promoters (Figure 3A). When 5-aza was used to inhibit gene methylation, TGF-β induced the down regulation of both TET2 and TET3, which were largely inhibited at the mRNA and protein levels (Figure 3B and 3C). In the metastatic melanoma cell line, SK-MEL-1, 5-aza treatment up regulated TET2 and TET3 expression levels (Figure 3D), which indicates that TET2 and TET3 are silenced through DNA methylation. Additionally, the switch from E-cadherin to N-cadherin and the enhancement of cell mobility induced by TGF-β1 were significantly inhibited by 5-aza treatment (Figure 3E and 3F). These data suggest that the inactivation of TET2 and TET3 induced by TGF-β1 is associated with the function of DNA methylation enzymes.

### TGF-β1 enhances the recruitment of DNMT3A to TET2 and TET3 promoters

**Figure 4:**
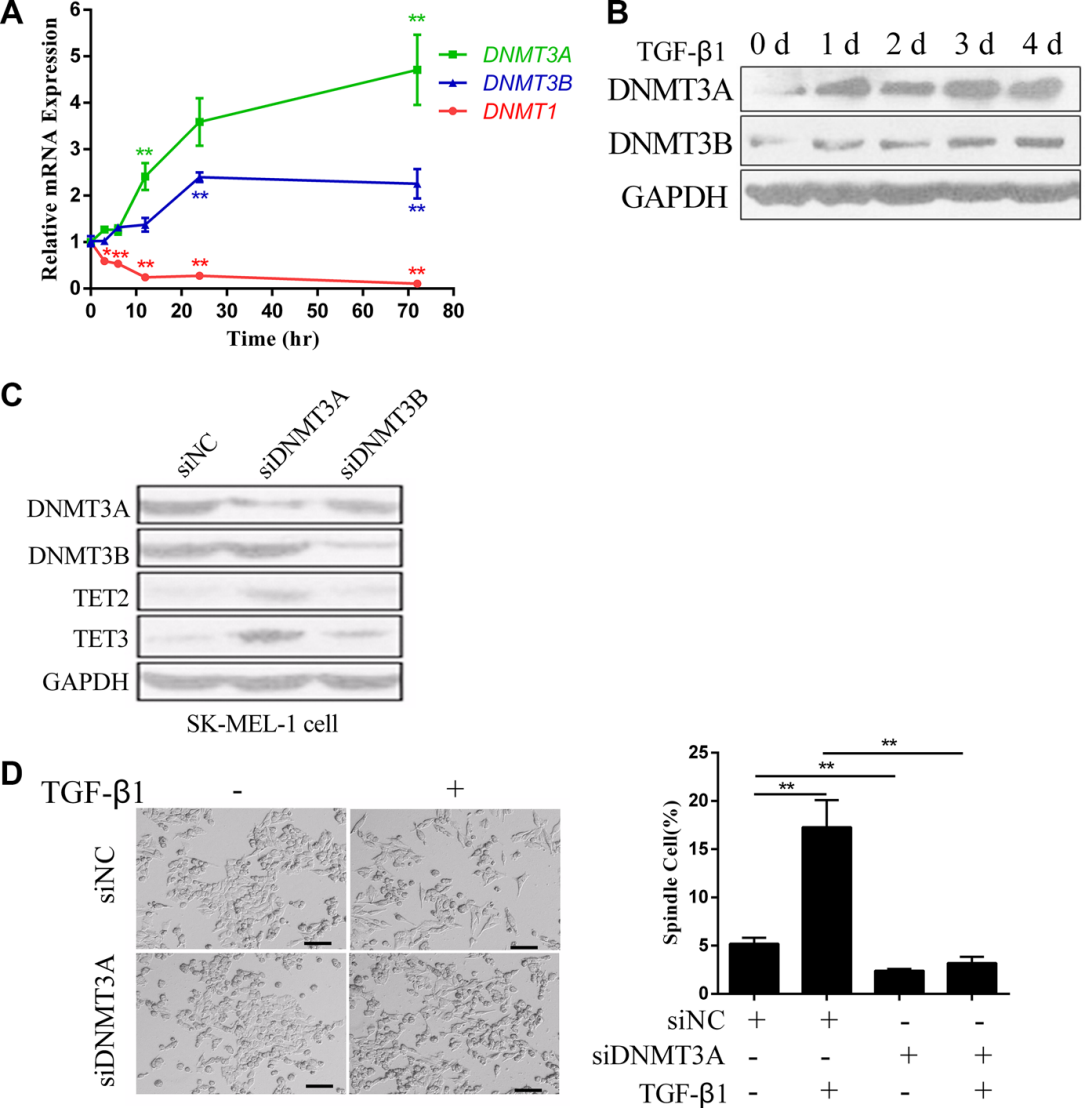
DNMT3A mediates the TGF-β1-induced down regulation of TET2 and TET3 (**A**) RT-qPCR and (**B**) immunoblotting analysis of DNMT1, DNMT3A and DNMT3B mRNA and protein levels in cells treated with or without TGF-β1 for the indicated times. For RT-qPCR, the relative expression levels of all genes were normalized to the level of *GAPDH* (Student's *t* test, *p < 0.05, **p < 0.01), while for immunoblotting, GAPDH was used to show that equal amounts of proteins were loaded on the gel; (**C**) The expression levels of TET2 and TET3 upon siRNA-mediated knock down of DNMT3A or DNMT3B in SK-MEL-1 cells were detected by immunoblotting, and GAPDH was used to show that equal amounts of proteins were loaded on the gel; (**D**) Representative morphology of A375 cells that were transfected with siRNAs against Negative Control (NC) or DNMT3A and that were treated with or without TGF-β1 are shown on the left (Left, Scale bar = 100 μm). The percentage of spindle-shaped cells was determined by counting the cells in 4 fields and is shown on the right (Student's *t* test, **p < 0.01).

DNMT1 (DNA Methyltransferase 1) is a maintenance methyltransferase that preserves the methylation status of newly synthesized DNA during cell cycles [21]. DNMT3A (DNA Methyltransferase 3A) and DNMT3B (DNA Methyltransferase 3B) are *de novo* DNA methyltransferases that catalyze DNA methylation at unmethylated genomic sites [21]. After 3 days of treatment with TGF-β1, the expression levels of DNMT3A and DNMT3B were significantly up regulated, while DNMT1 was down regulated (Figure 4A and 4B). This suggested that DNMT3A or DNMT3B might be repressors of TET2 and TET3. Therefore, we used siRNAs to knock down DNMT3A or DNMT3B in SK-MEL-1 cells, and found that the expression levels of TET2 and TET3 were up regulated upon knockdown of DNMT3A, but not after knock down of DNMT3B (Figure 4C). Moreover, the knockdown of DNMT3A significantly inhibited the EMT-like process induced by TGF-β1, as shown by the absence of morphological changes (Figure 4D). This result indicates that DNMT3A is responsible for the silencing of TET2 and TET3.

**Figure 5:**
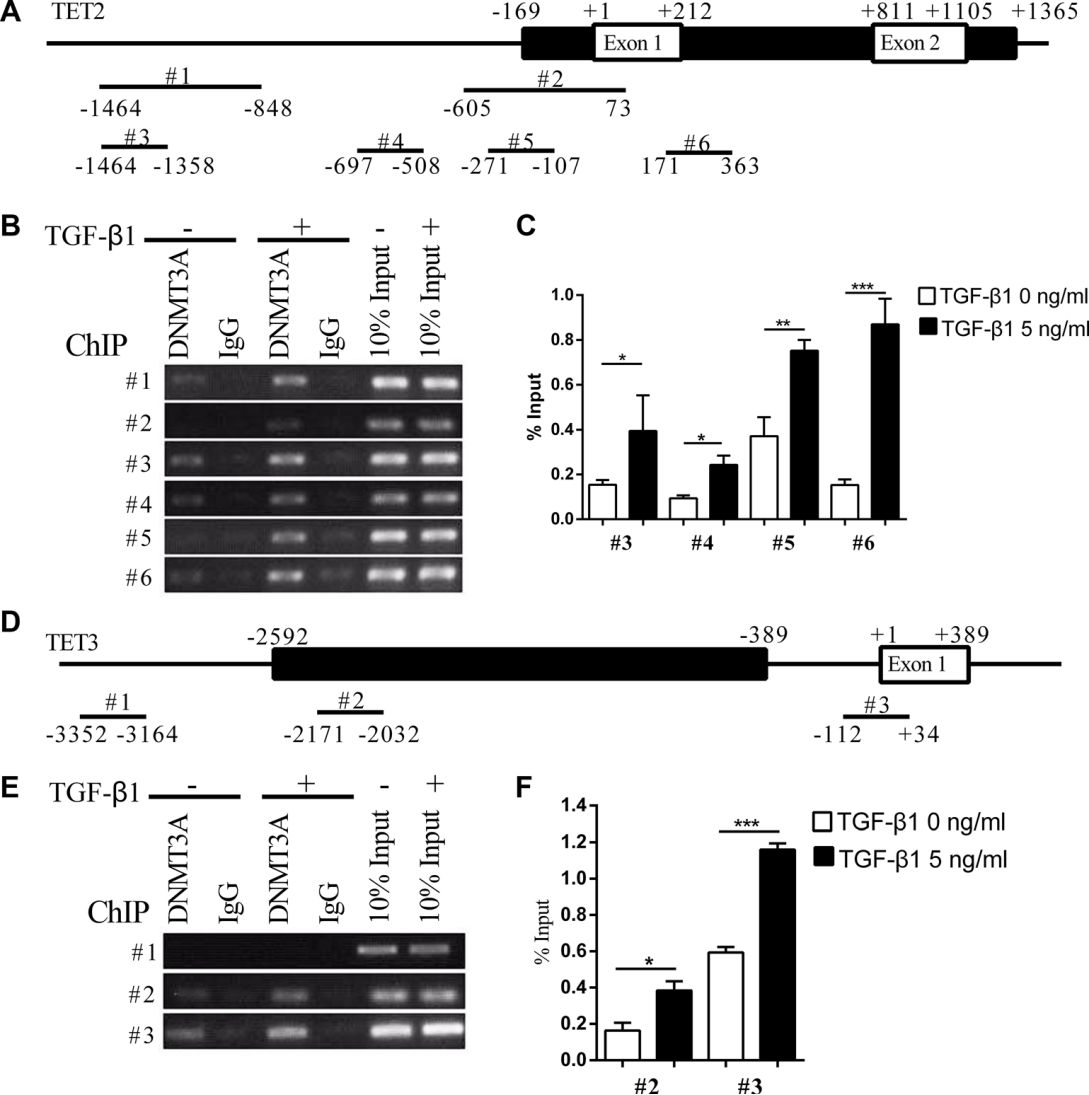
TGF-β1 enhances the recruitment of DNMT3A to the TET2 and TET3 promoters (**A**) and (**D**) show the schematic representation of the CpG islands near the TET2 and TET3 transcription start sites, respectively. The black lines indicate the CpG islands and the white frames indicate the exons, the position of the primers is indicated with numbers above, while the relative distances to the transcription start sites are marked below; (**B**), (**C**), (**E**) and (**F**) show the recruitment of DNMT3A to the TET2 and TET3 promoters with or without TGF-β1 treatment, which was analyzed by ChIP-PCR (B) (E) and ChIP-qPCR (C) (F) (Student's *t* test, *p < 0.05, **p < 0.01, ***p < 0.01).

We further analyzed whether DNMT3A physically interacts with the promoters of *TET2* and *TET3*. The CpG island within the *TET2* promoter spans the first two exons. It starts from 169 nt ahead of TSS (transcription start site) and ends at 1365 nt downstream of TSS (Figure 5A). ChIP-PCR showed that the treatment of the cells with TGF-β1 enhanced the recruitment of DNMT3A to the *TET2* promoter (Figure 5B and 5C). The CpG island near the *TET3* TSS starts at −2592 and ends at −389 (Figure 5D). ChIP-PCR showed that TGF-β1 treatment enhanced the recruitment of DNMT3A to *TET3* TSS and the CpG island but not the upstream sequence (Figure 5E and 5F). These data indicate that TGF-β1 treatment activated the recruitment of DNMT3A to *TET2* and *TET3* promoters.

### Overexpression of the TET2 C-terminal sequence inhibits the TGF-β1-induced EMT-like process

The results described above suggested that TET2 or TET3 might be a suppressor of the EMT-like process. The correlation between the expression levels of TET2/3 and EMT marker genes in 6 cell lines was further examined, and the results showed that the protein expression levels of TET2 were positively correlated with that of E-cadherin (Supplementary Figure S2). We also studied human melanoma datasets from The Cancer Genome Atlas (TCGA), and found that the TET proteins, especially TET2 were downregulated, and the mesenchymal marker Vimentin and EMT master transcription factors like *ZEB2*, *SNAIL2* and *TWIST1* were up regulated (Supplementary Figure S2C). Among the 474 samples, the expression levels of TET2 and TET3 were negatively correlated with Vimentin (data not shown).

**Figure 6:**
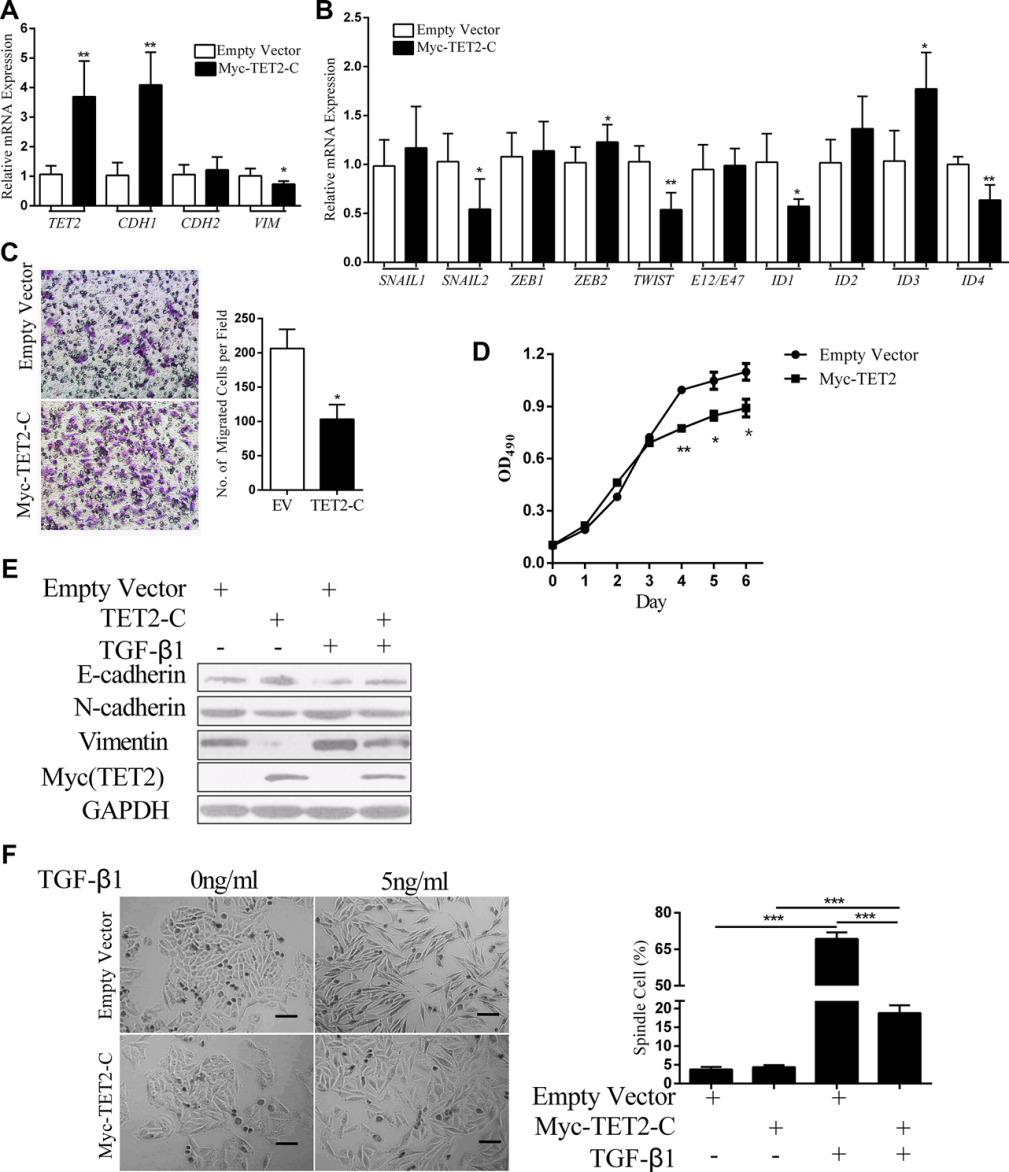
Overexpression of TET2 antagonizes TGF-β1-induced EMT (**A**, **B**) The relative expression levels of the indicated mRNAs in A375 cells that were transfected with empty vector or the TET2 C-terminal expression vector were detected by RT-qPCR, and all genes were normalized to the levels of *GAPDH*, (Student's *t* test, *p < 0.05, **p < 0.01); (**C**) Boyden Chamber Transwell cell migration assay. In all, 5 × 10^4^ cells were seeded on top of the Boyden chambers. After 24 hrs, the cells on the bottom were stained with 1% crystal violet and were observed by optical microscopy. Cell migration was determined by counting the number of stained cells; representative images of the migrated cells are shown on the right. The Y-axis represents the number of cells per field. The data represent three independent experiments (Student's *t* test, *p < 0.05, **p < 0.01); (**D**) 1 × 10^3^ cells were seeded into each well of 96-well plates, and cell growth was evaluated by measuring the absorbance of OD_490_ after staining with MTT. (**E**) The expression levels of the indicated proteins in A375 cells that were transfected with empty vector or TET2 expression vector and that were treated with or without TGF-β1 were detected by immunoblotting, and GAPDH was used to show that equal amounts of proteins were loaded on the gel; (**F**) Morphology of A375 cells that were transfected with empty vector or with the TET2 expression vector and that were treated with or without TGF-β1 for 3 days. Representative images of cell morphology are shown on the left (Scale bar =100 μm), and the percentages of spindle-shaped cells were derived from counts of 4 fields and are shown on the right; each experiment was repeated 3 times (Student's *t* test, ***p < 0.001).

To validate the functional role of TET2, the TET2 C-terminal sequence responsible for the catalytic process was overexpressed in A375 cells. The overexpression efficiency of TET2 was confirmed by real-time RT-PCR and immunoblotting (Figure 6A and 6E). The results showed that ectopic expression of the TET2 C-terminal sequence up regulated the expression of *CDH1*, down regulated the expression of *VIMENTIN*, decreased expression of the master EMT regulators *SNAIL2*, *TWIST*, *ID1* and *ID4* (Figure 6B) and inhibited cell migration and proliferation (Figure 6C, 6D). In response to TGF-β1, cells carrying the empty vector displayed mesenchymal morphology, whereas most of the cells that stably expressed the TET2 C-terminal domain retained an epithelial morphology (Figure 6F). TET2 overexpression also blunted TGF-β1 in inducing the down regulation of E-cadherin and the up regulation of N-cadherin and Vimentin (Figure 6E). Thus, our overexpression study also showed that TET2 is a suppressor of the EMT-like process.

### Overexpression of TET2 suppresses tumor growth and metastasis *in vivo*

**Figure 7:**
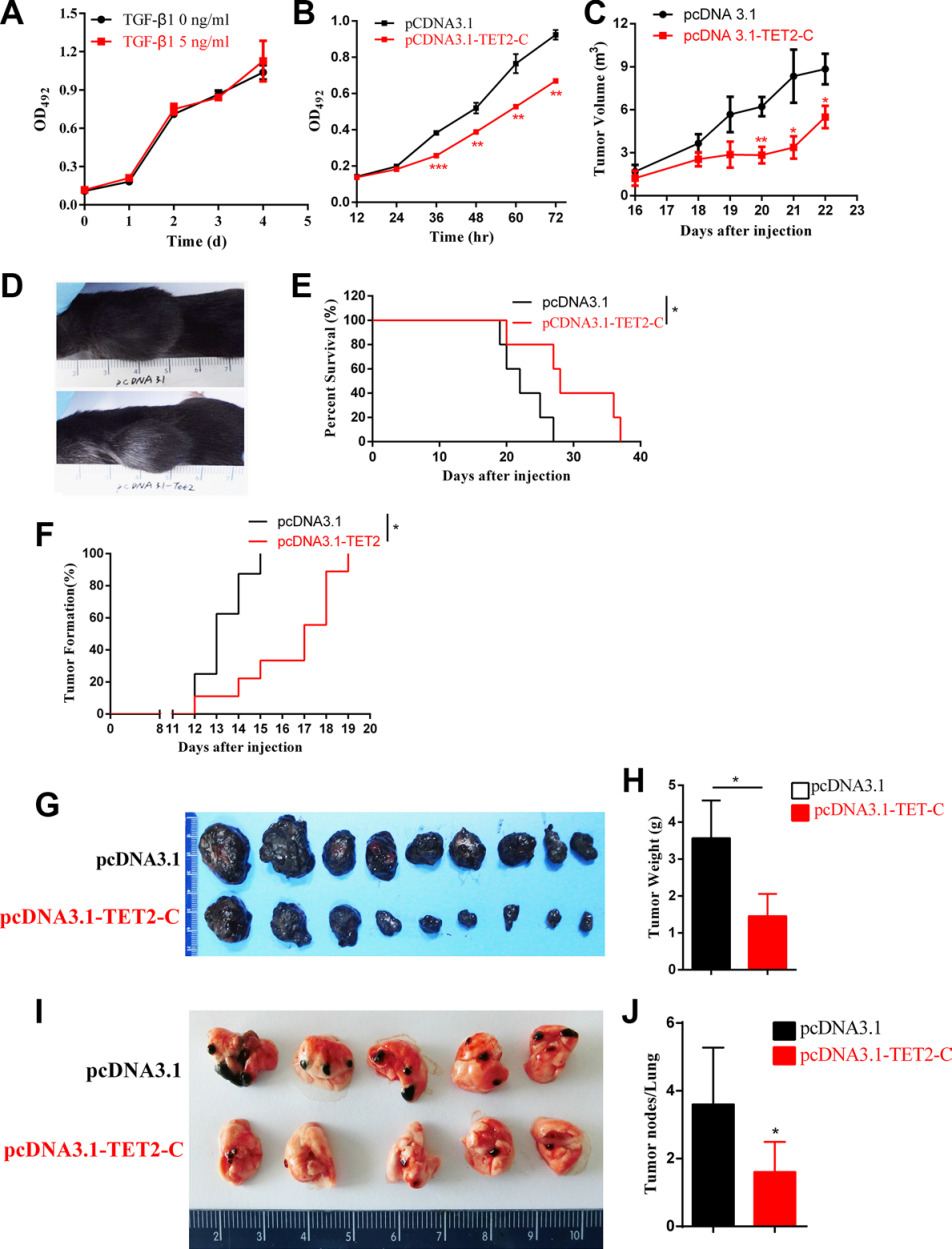
Overexpression of TET2 suppresses tumor growth and metastasis *in vivo* (**A**) The effect of TGF-β treatment on B16 cell growth was evaluated by measuring the absorbance of OD_490_ after staining with MTT. (**B**) TET2 C-terminal overexpression cells were transfected with the pcDNA3.1-TET2 C-terminal sequence or with pcDNA3.1 empty vector. The effect of TET2 overexpression on cell proliferation was assessed by MTT; 5 × 10^6^ cells were subcutaneously injected into the flanks of C57BL/6 mice (*n* = 5), and the volumes of the tumors were measured since 16 days after injection. A significant difference was observed after day 20 (**C)**, the volumes of the tumors were evaluated as lengths × widths^2^/2, and a representative image of the mice at day 22 is shown (**D**), Survival time of the mice is shown as the percentage of mice still alive at different times post injection (**E**); 1 × 10^6^ B16 cells transfected with pcDNA3.1-TET2 or empty vector were injected into the flanks of C57BL/6 mice, and the time of forming visible tumors was recorded (**F**). All mice were sacrificed and dissected 30 days after injection. The morphology of the tumors (**G**), the weights of the tumors (**H**) were shown; 2 × 10^5^ cells transfected with pcDNA3.1-TET2 or empty vector were intravenously inoculated into C57BL/6 mice (*n* = 9), and the mice were sacrificed on day 25 to evaluate the occurrence of metastasis by counting the number of tumor nodules in the lungs. The photograph of the lungs is shown (**I**). The metastatic potential of the cells was assessed by counting the number of tumor nodules in the lungs (**J**). (Student's *t* test, *p < 0.05, **p < 0.01, ***p < 0.001)

B16 cell is a murine tumor cell line with high metastatic potential. It is frequently used in melanoma models. The proliferation of B16 cell is insensitive to TGF-β in culture (Figure 7A). We then established B16 cell lines that were stably transfected with pcDNA3.1-TET2 C-terminal or empty vector, and found that the over expression of the TET2 C-terminal domain decreased cell proliferation *in vitro* (Figure 7B). We then established xenograft models via subcutaneous injection of 5×10^6^ cells into the flanks of C57BL/6 mice to test the effects of TET2 expression on tumor growth and survival of the mice. Notably, the overexpression of TET2 significantly reduced tumor formation as indicated by reduced tumor volume and longer survival time of the mice (Figure 7C, 7D and 7E). In another experiment, 1 × 10^6^ cells were injected as in the previous experiment. The overexpression of the TET2 C-terminal sequence significantly delayed the appearance of macroscopic tumors (Figure 7F) and decreased the tumor weights (Figure 7G and 7H) by 35 days after injection.

To explore the potential role of TET2 in melanoma metastasis *in vivo*, 1 × 10^5^ cells were intravenously injected into C57BL/6 mice to generate a mouse model of pulmonary metastatic melanoma. We observed that overexpression of the TET2 C-terminal sequence significantly decreased the metastatic potential as evidenced by the reduced number of tumor nodules in the lungs at day 25 (Figure 7I and 7J).

Taken together, the above data show that the overexpression of TET2 significantly inhibits the EMT-like process of melanoma cells *in vitro* and suppresses tumor growth and metastasis *in vivo*.

## DISCUSSION

**Figure 8:**
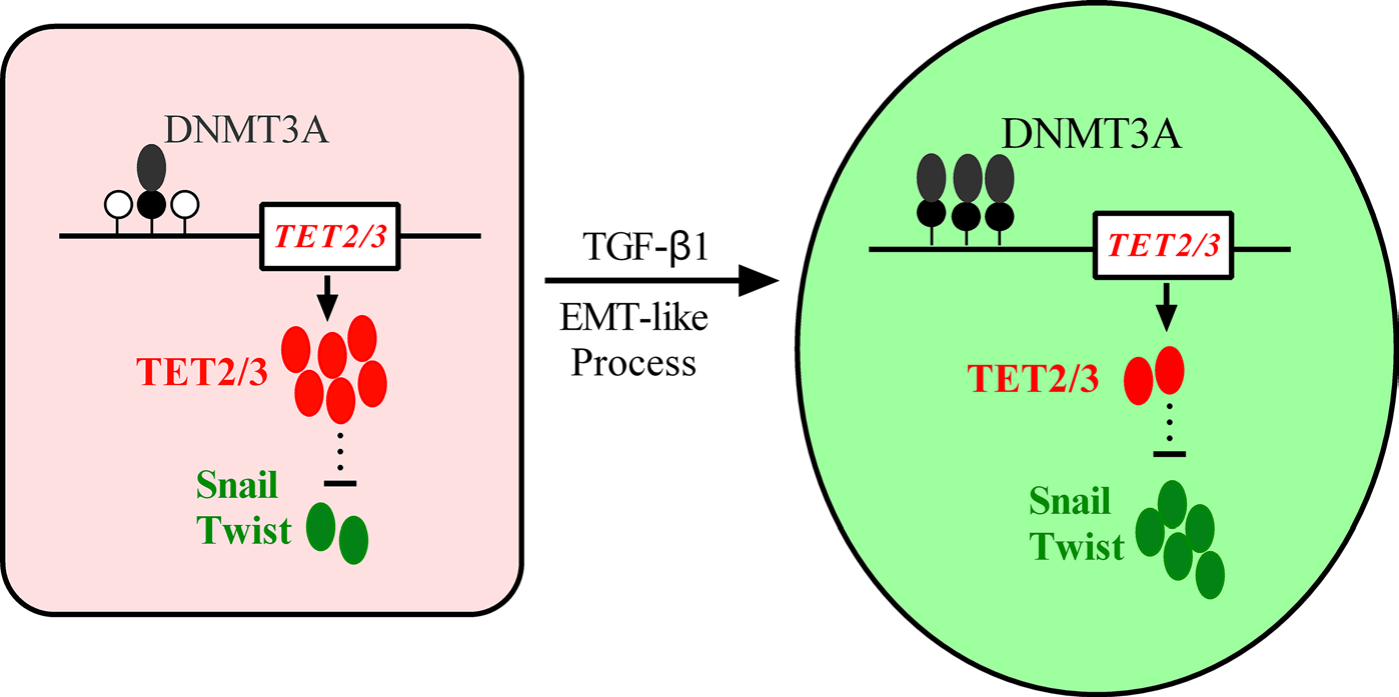
A model depicting the roles of epigenetic silencing of TET2 and TET3 in TGF-β-induced EMT-like process in melanoma TGF-β treatment enhanced the recruitment of DNMT3A to TET2 and TET3 promoters, and the silencing of them activated EMT master transcription factors to promote the EMT-like process in melanoma.

Malignant melanoma represents a cancer that has continued to increase in incidence worldwide [22]. Aberrant DNA methylation profiles have been reported in many cancers [23, 24]. TGF-β1 induces global changes in DNA methylation during EMT in ovarian cancer cells [25, 26]. The TET protein family, which catalyzes active DNA demethylation, has been reported to be lost in melanoma [19]. Here, we found that TET2 and TET3 play critical roles in the EMT-like process that occurs in melanoma. These two proteins are down regulated through DNMT3A-mediated epigenetic silencing in the TGF-β1-induced EMT-like process. The knock down of TET2 or TET3 induces this EMT-like process. In addition, the overexpression of the TET2 C-terminal sequence partially rescues the TGF-β1-induced EMT-like process *in vitro* and inhibits tumor growth and metastasis *in vivo*. Hence, our data suggest an epigenetic circuitry that mediates the EMT activated by TGF-β (Figure 8). As an effector, DNMT3A senses the TGF-β signal and modifies TET2 and TET3 promoters to induce the EMT-like process and metastasis in melanoma.

The expression levels of TET genes are regulated by several mechanisms. *TET* genes are transcriptionally regulated by Oct4 (Octamer-binding Transcription Factor 4) in embryonic stem cells [27]. *TET* mRNAs are targeted by miR-22 in breast cancer [28] and an extensive network of microRNAs in malignant hematopoiesis [29]. The stability of TET proteins are regulated by caspases [30] and calpains [31]. Here, our results indicate that TET2 and TET3 are epigenetically silenced by DNMT3A-catalyzed DNA methylation in the TGF-β1-induced EMT-like process in melanoma.

EMT, a developmental program, is reactivated during carcinogenesis [4]. For example, Thrombospondin 1 was reported to promote an aggressive phenotype through inducing EMT in melanoma [32]. In addition to transcriptional regulation induced by the classical EMT transcription factors, dynamic epigenetic changes also occur to regulate the expression of both epithelial and mesenchymal genes [33]. TET proteins, which are responsible for active DNA demethylation, are found here to be critical regulators of the EMT-like process in melanoma cells. As DNA methylation is usually responsible for transcriptional repression, the down regulation of TET proteins would in turn lead to the hypermethylation of downstream genes, some of which may release the expression of master EMT transcription factors and thus activate the EMT-like process. In fact, the overexpression of TET2 has been reported to induce higher 5-hmC levels in genes that are associated with cancer-related pathways, such as genes that code for focal adhesion and adherens junction proteins [19]. It will be interesting if, in the future, investigators dissect the signals that mediate the involvement of TET2 and TET3 in the regulation of the EMT-like process in melanoma.

According to the widely described “phenotype-switching” model, the transition from proliferative phenotype cells to invasive phenotype cells, which is similar to the EMT, contributes to the progression of melanoma [2]. In our experiments, overexpression of TET2 C-terminal partially suppressed the *in vivo* EMT-like process and the *in vivo* metastasis, yet it inhibited *in vitro* cell proliferation and the *in vitro* melanoma growth. This suggests that alternations of the TET proteins expression levels could not fully bridge the “phenotype-switching” theory and the EMT-like process.

TET1 was reported to be a suppressor of KRAS-mediated transformation [34]. It also suppresses breast cancer invasion via the activation of the tissue inhibitors of metalloproteinases [35]. However, TET1 is up regulated by hypoxia and functions as a co-activator in the regulation of EMT and in gene expression as part of the hypoxia-response program [36]. TET1 is also frequently activated in mixed lineage leukemia (MLL) [37]. Here, in the TGF-β1-induced EMT-like process in melanoma, only TET2 and TET3 are down regulated. This indicates that the regulation of *TET* genes is likely dependent on the tissue types or the different conditions that induce EMT. Furthermore, the roles of TET proteins in the regulation of cancer progression are probably different in that TET1 is primarily involved in tumor initiation while TET2 and TET3 are primarily involved in cancer metastasis.

In summary, our *in vitro* experiments showed that TET2 and TET3 are suppressors of the EMT-like process in melanoma and DNMT3A-mediated epigenetic silencing is one mechanism by which TGF-β induces an EMT-like process. Our *in vivo* experiment showed that the overexpression of TET2 inhibits the growth and metastasis of melanoma. Hence, these experiments emphasize that the epigenetic silencing of TET2 and TET3 critically contributes to the progression of melanoma and may help provide additional pertinent information for cancer diagnosis and treatment.

## MATERIALS AND METHODS

### Animals

C57BL/6 mice were purchased from the Weitonglihua Company, Beijing, China, and were maintained under specific pathogen-free conditions. The mice were housed in the vivarium of the Institute of Genetics and Developmental Biology, Chinese Academy of Sciences. All procedures were approved by the Institutional Animal Care and Use Committee of the Institute of Genetics and Developmental Biology, Chinese Academy of Sciences. All animals were male and were 8~10 weeks old. In all, 5 × 10^6^ or 1 × 10^6^ B16 cells in 100 μl of phosphate-buffered saline (PBS) were subcutaneously injected into the flanks of the mice to test tumor growth and survival of the mice. Additionally, 1 × 10^5^ cells were intravenously injected into the mice to generate a model of pulmonary metastatic melanoma. The mice were sacrificed at the indicated time points, and the tumors were excised and weighed.

### Cell culture

A375, M619, SK-MEL-28, A875, B16 and SK-MEL-1 cells were purchased from the Cell Center of the Chinese Academy of Medical Sciences. A375, M619, A875 and B16 cells were cultured in Dulbecco's Modified Eagle's Medium (DMEM), SK-MEL-28 cells were cultured in RPMI 1640 medium, and SK-MEL-1 cells were cultured in MEM medium. All media were supplemented with 10% fetal bovine serum (FBS), 100 U/ml penicillin and 100 μg/ml streptomycin. All cells were cultured in a humidified 5% CO_2_ incubator at 37°C. Stable cell lines were selected and maintained in 500 mg/ml G418.

### Antibodies and reagents

The goat anti-TET1 (SC-163443), rabbit anti-TET3 (SC-139186), rabbit anti-DNMT3A (SC-20703), and rabbit anti-GAPDH (SC-25778) antibodies were all purchased from Santa Cruz Biotechnology (Santa Cruz, USA). Mouse anti-TET2 (61389) was purchased from Active Motif, while the mouse anti-E-cadherin (5296S) and rabbit anti-N-cadherin (4061S) antibodies were purchased from Cell Signaling Technology. Recombinant mature human TGF-β1 was purchased from R&D Systems (R&D Systems Inc., Minneapolis, MN, USA), and the cells were treated at the concentration of 5 ng/ml in the culture medium to induce EMT. The pan DNA methylation inhibitor 5-aza (5′-aza-2′-deoxycytidine; Sigma-Aldrich, Sweden, AB) was dissolved in 50% acetic acid and was further diluted in serum-free medium before incubation with the cells for the indicated time periods. The siRNA antisense sequences were as follows: DNMT3A-AS (5′-CAGGAGATGATGTCCAACCC-3′) and DNMT3B-AS (5′-CGTCGTGGCTCCAGTTACAA-3′).

### Plasmid construct

The human TET2 C-terminal sequence was cloned and inserted into pcDNA 3.1 expression vector using the following primers: 5′-CTCTAGACTCGAG CGATGGATTTCCCATCTTGCAGATG-3′ (Xho1) and 5′-CTTGGTACCGAGTATATATCTGTTGTAAGG-3′ (Kpn1).

### Wound healing assay

The cells were grown to confluence in complete cell culture medium. At time 0, a 3-mm scrape wound was created across the diameter with a pipette tip, which was followed by extensive washes with medium to remove dead and floating cells. Cell migration was recorded at the indicated time. Images were captured using an inverted microscope equipped with a digital camera and were later analyzed after determination of the distance between the cells on either side of the scratch over time. Wound closure was monitored by microscopy at × 100 magnification.

### Boyden chamber cell migration assays

In all, 30,000 cells in serum-free media were seeded into the Transwell inserts containing 8-μm permeable pores and were allowed to migrate toward 10% FBS-containing medium. Then, 24 or 48 hours later, the cells in the Transwell inserts were removed, and the inserts were washed in PBS three times. The migrated cells on the bottom of the insert were fixed in 2% glutaraldehyde solution followed by crystal violet (1%) staining. After the inserts were washed three times with PBS, the inserts were allowed to air dry, and images were obtained using an inverted microscope. Four independent fields were counted for each Transwell insert, and the average number of cells per field is represented in the graphs.

### MTT assay

B16 cells at a density of 1 × 10^3^ per well were seeded into a 96-well plate in replicates of 6, and cell proliferation was measured over the indicated time. Absorbance at 490 nm was read using a PerkinElmer EnSpire multimode reader (PerkinElmer, Waltham, MA, USA).

### Real-time PCR

Total RNA was isolated using TRIzol reagent (Invitrogen, USA). First-strand cDNA synthesis was performed using PrimeScript RT Master Mix (TaKaRa Biotech, Dalian, China). The mRNA levels of genes of interest were measured by real-time PCR using SYBR Green Master Mix (TaKaRa Biotech, Dalian, China). The total amount of mRNA was normalized to endogenous GAPDH levels.

### ChIP-PCR

Following TGF-β1 treatment, cell lysates were prepared. Chromatin was sonicated to obtain fragmented DNA (200–500 bp), which was then immune-precipitated with control IgG or with anti-DNMT3A. Precipitated DNA was measured by real-time PCR using SYBR Green Master Mix.

### Statistical analyses

The paired two-tailed Student's *t-test* was used for comparisons between the two groups. Statistical significance was defined as *p < 0.05, **p < 0.01, and ***p < 0.001. Pearson correlation analysis was used to evaluate the correlation between the expression levels of different proteins.

## SUPPLEMENTARY MATERIALS



## Abbreviations

The abbreviations used are

TET: ten-eleven-translocation 5-methylcytosine dioxygenases
EMT: Epithelial-Mesenchymal Transition
DNMT3A/B: DNA Methyltransferase 3A/B
TGF-β: Transforming Growth Factor-β
ZEB: zinc finger E-box binding homeobox
bHLH: basic helix–loop–helix
ID: inhibitor of differentiation
