# Supplementary material for: Epigenetic silencing of TET2 and TET3 induces an EMT-like process in melanoma
Source: Oncotarget. 2016 Nov 12;8(1):315–28. doi: 10.18632/oncotarget.13324 (PMC5352122; doi:10.18632/oncotarget.13324)
Supplement: Supplementary file 1 [file oncotarget-08-315-s001.pdf]

Epigenetic silencing of TET2 and TET3 induces an EMT-like process in melanoma

Supplementary Materials

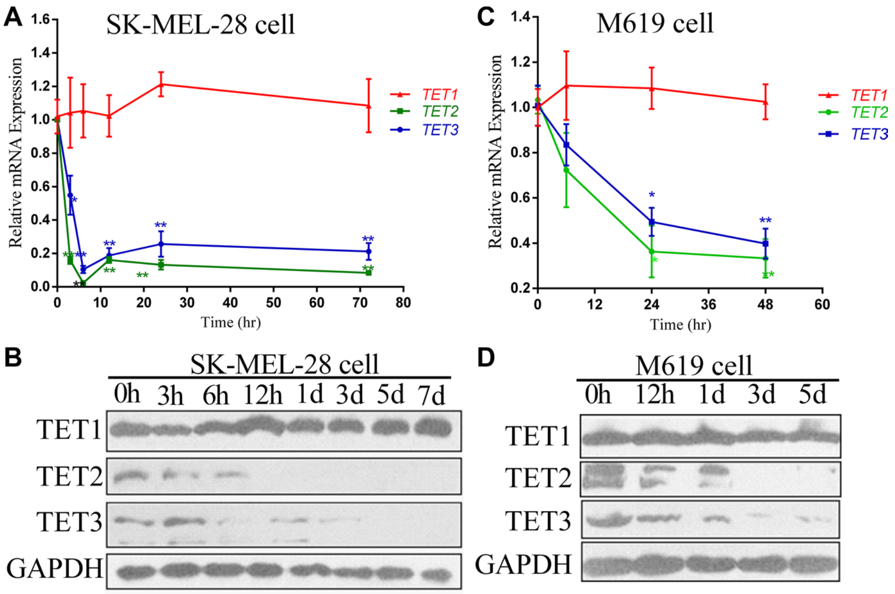

Supplementary Figure S1: TGF-β1 induces the down regulation of TET2 and TET3 in SK-MEL-28 cells and M619 cells. The expression levels of TET1, TET2 and TET3 mRNA and protein in SK-MEL-28 cells and M619 cells cultured with TGF-β1 for different times were measured by RT-qPCR (**A**–**C**) and immunoblotting (**B**–**D**). For RT-qPCR, the data are displayed as the fold change in the expression levels in untreated cells and were normalized by *GAPDH* (Student's *t* test, $*p < 0.05$, $**p < 0.01$); for immunoblotting, GAPDH was used to show that equal amounts of proteins were loaded on the gel.

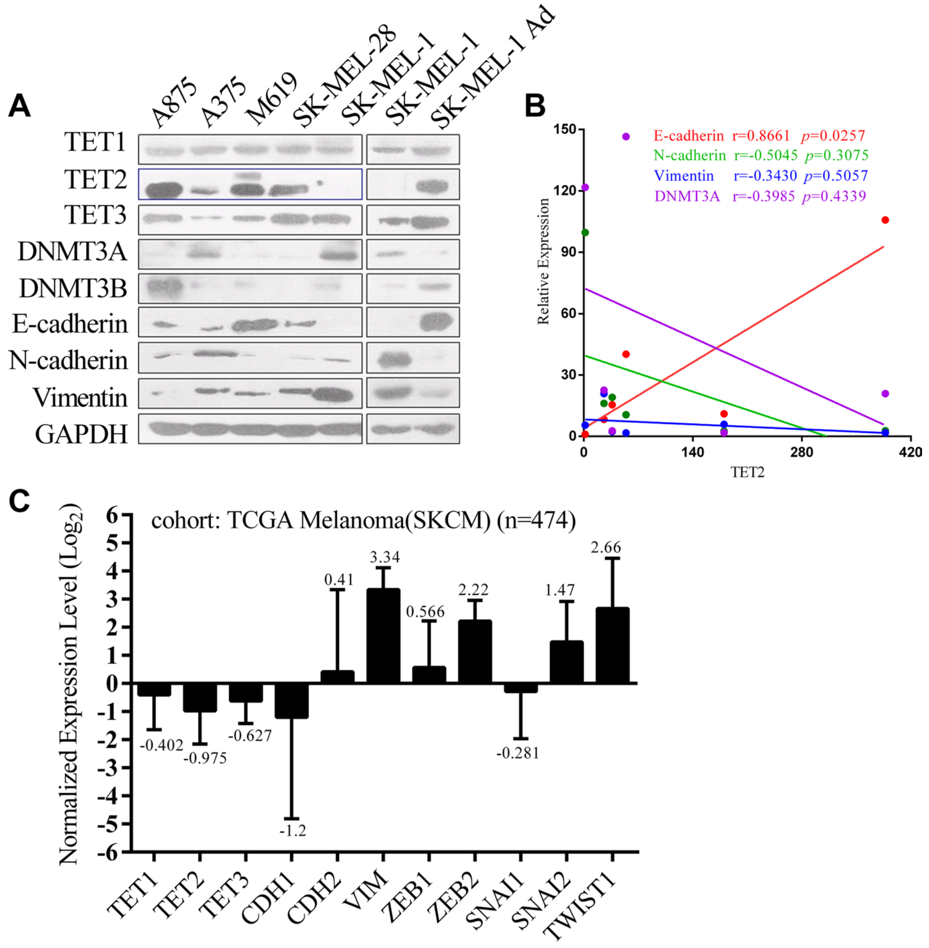

Supplementary Figure S2: Expression of the TET protein family in different cell lines and human melanomas. (**A**) The expression levels of the indicated proteins were detected in 6 different melanoma cell lines by immunoblotting, and GAPDH was used to show that equal amounts of proteins were loaded on the gel; (**B**) Scatter plots show the Pearson correlations of E-cadherin, N-cadherin, Vimentin, the DNMT3A levels and the TET2 level. The Y-axis represents the relative expression levels of the indicated genes in different cell lines, while the X-axis represents the relative expression levels of TET2 in different cell lines. Correlations (r) and corresponding *p*-values are indicated in different colors: $r > 0$ indicates a positive correlation, $r < 0$ indicates a negative correlation, and $p < 0.05$ is considered a significant correlation; (**C**) The expression levels of TET gene family and EMT related genes of TCGA melanoma comprising 474 samples. The X-axis represents different genes and the Y-axis represents the normalized expression levels (Log_2) of the indicated genes.
